# Complete Genome Sequence of *Cycloclasticus* sp. Strain PY97N, Which Includes Two Heavy Metal Resistance Genomic Islands

**DOI:** 10.1128/MRA.00771-19

**Published:** 2019-10-03

**Authors:** Zhisong Cui, Veronika Kivenson, Na Liu, Angela Xu, Xiao Luan, Wei Gao, Blair Paul, David L. Valentine

**Affiliations:** a Key Laboratory for Marine Bioactive Substances and Modern Analytical Technology, First Institute of Oceanography, Ministry of Natural Resources of China, Qingdao, China; b Department of Earth Science and Marine Science Institute, University of California, Santa Barbara, Santa Barbara, California, USA; c Interdepartmental Graduate Program in Marine Science, University of California, Santa Barbara, Santa Barbara, California, USA; d College of Creative Studies Biology, University of California, Santa Barbara, Santa Barbara, California, USA; e State Key Laboratory of Environmental Aquatic Chemistry, Research Center for Eco-Environmental Sciences, Chinese Academy of Sciences, Beijing, China; University of Arizona

## Abstract

We present the complete genome sequence of fluoranthene-consuming Cycloclasticus sp. strain PY97N. This strain has one circular chromosome with a G+C content of 42.06%. Moreover, two genomic islands were identified as putative conjugative elements. These genomic details are expected to inform our understanding of the remarkable catabolic capacities of organisms of the *Cycloclasticus* lineage.

## ANNOUNCEMENT

Strains of Cycloclasticus, either free-living or symbiotic, play a pivotal role in the hydrocarbon removal process. *Cycloclasticus* sp. strain PY97N, isolated from Yellow Sea sediment, is specialized with a novel catabolic characteristic to consume fluoranthene (1–5). Here, we investigated the genome of strain PY97N.

The general features of strain PY97N are summarized in Table 1. A glycerol stock of strain PY97N was inoculated into ONR7a medium (6) supplemented with 0.2 g/liter phenanthrene as a sole source of carbon at a proportion of 0.1% (vol/vol); the culture was then kept under 150 rpm at 25°C until late-logarithmic phase for cell harvesting. The genomic DNA was then extracted using the AxyPrep bacterial genomic DNA miniprep kit (Axygen Scientific, USA). The whole-genomic DNA sequencing was performed using a combination of the PacBio RS II and Illumina HiSeq 4000 platforms at the Beijing Genomics Institute (Shenzhen, China). The PacBio library, with an average insert size of 11 kb, generated 94,155 reads totaling 1,236,526,834 bp. This sequencing provided 509-fold coverage of the genome, which enables the assembly of a high-quality circular genome. SMRT Analysis v2.3.0 was then applied for assembly (7). Default parameters were used for all software, unless otherwise specified. Moreover, the Illumina library, with an average insert size of 500 bp, generated 3,806,998 paired-end reads totaling 448,000,000 bp, and the next-generation sequencing (NGS) data were utilized to correct the single base error in the assembly result by the GATK v1.6-13 (https://software.broadinstitute.org/gatk/) and SOAP (8, 9) tool packages.

**TABLE 1:** General features of *Cycloclasticus* sp. PY97N and MIGS mandatory information^*a*^

General feature	Description
Classification	
Domain	*Bacteria*
Phylum	*Proteobacteria*
Class	*Gammaproteobacteria*
Order	*Thiotrichales*
Family	*Piscirickettsiaceae*
Genus	*Cycloclasticus*
Species	*Cycloclasticus* sp.
Gram stain	Negative
Cell shape	Rod
Motility	Motile
Pigmentation	Nonpigmented
Sporulation	Nonsporulating
Growth temp (°C)	20
Carbon source	Polycyclic aromatic hydrocarbons
Energy source	Chemoorganotrophic
Terminal electron receptor	Oxygen
Salinity (‰)	36
Oxygen	Aerobic
MIGS data	
Submitted to INSDC^*b*^	GenBank accession no. CP023664
Investigation type	Bacteria
Project name	*Cycloclasticus* sp. strain PY97N genome sequencing and assembly
Geographic location	
Latitude, longitude	36.67°N, 121.99°E
Depth (m)	17.8
Country	China (Yellow Sea)
Collection date (yr-mo-day)	2007-04-05
Environment	
Biome	Ocean
Feature	Sediment
Material	Sea sediment
Environmental package	Sea sediment samples from Yellow Sea
Biotic relationship	Free-living
Pathogenicity	None
Sequencing methods	PacBio RS II, Illumina HiSeq 4000
Assembly	RS_HGAP Assembly3 in SMRT Analysis v2.3.0
Finishing strategy	Complete

a MIGS, minimum information about a genome sequence.

b INSDC, International Nucleotide Sequence Database Collaboration.

Gene prediction was performed using Prodigal v.2.6.2 (10) with the single-genome mode, while tRNA genes were predicted with tRNAscan-SE v.1.3.1, and rRNA genes were predicted with rRNAmmer v.1.2 (11). The predicted open reading frames (ORFs) were used as input for an RPS-BLAST search against the NCBI Conserved Domain Database (CDD) and Cluster of Orthologous Groups (COG) database (12, 13). Moreover, the genome of strain PY97N was also annotated using PGAP (14).

The complete genome consists of one chromosome with a total length of 2,430,152 bp and a G+C content of 42.06%. Of the 2,367 genes predicted, 2,322 were protein-coding sequences (CDSs) and 45 were RNAs. Of the entire 2,322 CDSs, 1,928 could be assigned to COGs.

Genome analysis of strain PY97N reveals the existence of 8 genes for aromatic ring-hydroxylating dioxygenase alpha subunit and 13 genes of the beta subunit, 7 of which present pairwise.

**FIG 1:**
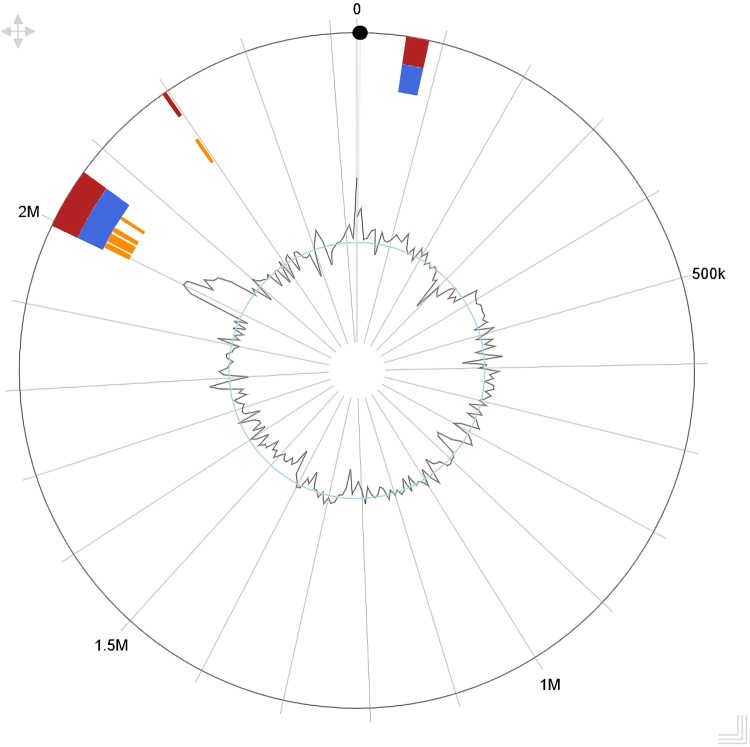
Putative genomic islands in *Cycloclasticus* sp. PY97N predicted by IslandViewer4. Two genomic islands (blue areas) were predicted by IslandViewer4 using the IslandPath-DIMOB method. The smaller island starts from 56701 and ends at 83656. The larger island starts from 1987924 and ends at 2065547. The orange areas are precise, yet confined, regions of genomic islands predicted by the SIGI-HMM method. The red areas show the prediction by both methods. The curve at the inner circle represents the GC content.

Two putative genomic islands encompassing at least 77,623 bp and 26,955 bp were predicted by the IslandViewer4 software using both the IslandPath-DIMOB and SIGI-HMM methods (15) (Fig. 1). They both had a relatively higher G+C content (48.80% and 42.64%, respectively) than the average G+C content of the genome. The two putative islands contained 74 and 28 genes, respectively. Genes that are commonly found in mobile genetic elements (MGEs), including transposase, integrase, and recombinase, were identified both at the beginning and end of the two islands, suggesting that these features can self-mobilize, possibly mediating horizontal gene transfer (HGT) in this strain. For the larger island, at least 24 genes (32% of the total) related to heavy metal utilization and resistance were annotated, including the regulatory system *cusS-cusR,* and genes *copA* and *copB,* encoding copper resistance proteins. These genes indicate a potential for tolerance for extreme copper stress (16, 17). We also found a complete *czc* efflux system which produces cobalt-zinc-cadmium resistance proteins.

### Data availability.

The complete genome sequence of *Cycloclasticus* sp. PY97N is available in GenBank under the accession number CP023664; both the PacBio reads and the Illumina reads are available in the SRA under accession number PRJNA411517.
